# Analysis of pathogenesis-related factors of *Streptococcus mutans* from the oral cavity of patients with nonalcoholic steatohepatitis

**DOI:** 10.3389/fimmu.2026.1879236

**Published:** 2026-10-14

**Authors:** Keiko Tabata, Shuhei Naka, Kana Goto, Shuichi Tonomura, Taro Misaki, Michiyo Matsumoto-Nakano

**Affiliations:** 1 Department of Pediatric Dentistry, Okayama University Graduate School of Medicine, Dentistry and Pharmaceutical Sciences, Okayama, Japan; 2 Department of Vascular Physiology, National Cerebral and Cardiovascular Center, Osaka, Japan; 3 Division of Nephrology, Seirei Hamamatsu General Hospital, Shizuoka, Japan; 4 Department of Nursing, Faculty of Nursing, Seirei Christopher University, Shizuoka, Japan

**Keywords:** adiponectin, collagen binding protein, non-alcoholic steatohepatitis, protein antigen, streptococcus mutans, Type IV collagen 7s

## Abstract

Non-alcoholic fatty liver disease (NAFLD) comprises non-alcoholic fatty liver (NAFL), which rarely progresses, and non-alcoholic steatohepatitis (NASH), which may progress to cirrhosis and hepatocellular carcinoma. Previous studies have shown that *Streptococcus mutans* positive for collagen-binding protein (Cnm) and the 190-kDa cell surface protein antigen (PA) may be involved in NASH progression. In this study, we analyzed saliva samples obtained from patients with NAFL and NASH, and investigated the involvement of these proteins in NASH development by administrating clinical isolates to a mouse model. There was no significant difference in the detection rate of *S. mutans* between the NAFL and NASH patient groups; however, Cnm^+^/PA^+^*S. mutans* strains were more frequently detected in patients with NASH. Furthermore, patients with NASH with Cnm^+^/PA^+^ *S. mutans* showed significantly higher levels of type IV collagen 7S, a biomarker of liver fibrosis, compared to patients with NASH without these strains. Adiponectin, an anti-inflammatory cytokine mainly secreted by adipocytes, was significantly lower. The collagen-binding ability of Cnm^+^/PA^+^ *S. mutans* strains was significantly higher than that of other *S. mutans* strains without both proteins. Cnm^+^/PA^+^ *S. mutans* showed significantly higher binding ability to type IV collagen than to type I collagen. RT-qPCR analysis revealed that *pac* expression was significantly higher in Cnm^+^/PA^+^ *S. mutans* isolated from the NASH group. Administration of Cnm^+^/PA^+^ *S. mutans* into a NASH mouse model via the jugular vein caused NASH-like pathology in mouse liver tissues. These results suggest that Cnm^+^/PA^+^ *S. mutans* is associated with NASH aggravation.

## 1 Introduction

*Streptococcus mutans* is a Gram-positive facultative anaerobic bacterium that is a major pathogen of dental caries. It is also occasionally isolated from extra-oral sites, which implies a possible association with systemic diseases such as infective endocarditis (IE), valvular disease, and cerebral hemorrhage (1–4). A critical step in the development of IE is bacterial attachment to the impaired endothelium. The 120-kDa collagen-binding protein Cnm of *S. mutans* is associated with not only collagen binding but also the adhesion to and invasion of artery and vein endothelial cells (5). Approximately 10%-20% of *S. mutans* strains in the human oral cavity express Cnm. The 190-kDa cell surface protein antigen PA is expressed in approximately 90% of clinical isolates of *S. mutans* (5). PA is associated with sucrose-independent initial adhesion of *S. mutans* to tooth surfaces and cellular hydrophobicity (6) and the release of various inflammatory cytokines (7). Approximately 7.5% of *S. mutans* is positive for both Cnm and PA (Cnm^+^/PA^+^) *S. mutans* is estimated to be approximately 7.5% (5).

Fatty liver disease is divided into alcoholic liver disease and non-alcoholic fatty liver disease (NAFLD). NAFLD is further classified into non-alcoholic fatty liver (NAFL) and non-alcoholic steatohepatitis (NASH) based on liver biopsy. NASH was first proposed in 1980 as a new disease concept in cases of unknown etiology with histopathological findings similar to alcoholic steatohepatitis with no apparent history of alcohol consumption (8). Without treatment, NASH can progress to cirrhosis and even hepatocellular carcinoma in severe cases (9). An early study showed that *S. mutans* aggravated NASH in specific model mice (10). Moreover, we found that Cnm^+^/PA^+^ *S. mutans* adhered to hepatocytes via Cnm and bound unsaturated fatty acids via PA, resulting in increased fibrosis; this mechanism may be involved in the pathological progression from NAFL to NASH (11). We are recently reported that *S. mutans* harboring Cnm was significantly more frequently detected in patients with NASH (12).

In this study, we used *S. mutans* strains isolated from patients with NAFLD to analyze the characteristics and molecular biological properties of Cnm and PA, thereby investigating their roles in the progression of NASH. We also evaluated disease progression associated with Cnm^+^/PA^+^ *S. mutans* isolated from patients with severe NASH using a mouse NASH model.

## 2 Materials and methods

### 2.1 Subjects and samples

This study included patients with NAFLD at Nara City Hospital. All patients provided written informed consent in accordance with the Declaration of Helsinki. The diagnosis of NASH was made as described in a previous study (12). Briefly, most patients underwent liver biopsy, and a pathological diagnosis of NASH was made based on the NAFLD activity score. For patients who did not undergo liver biopsy, conventional ultrasound was used was used to evaluate hepatic steatosis, and possible NASH was diagnosed using the NASH, ferritin, insulin, type IV collagen 7S (NAFIC) score using ferritin, fasting insulin, and type IV collagen 7S. Patients who withdrew after providing informed consent, those could not undergo oral sampling, and those with alcoholic hepatitis were excluded. The final group included 40 patients with NAFLD (NASH n=20, NAFL n=20). Blood samples were collected from the NAFLD patients. This study was approved by the Nara City Hospital Ethics Committee (Approval No; 32) and Okayama University Graduate School of Medicine, Dentistry and Pharmaceutical Sciences and Okayama University Hospital, Ethics Committee (Approval No; 1707-021).

*S. mutans* strains were used in this study isolated from the oral cavity of NAFLD patients described previously (12). Participants in this study were asked to visit the hospital at least 9 h after their last meal. The saliva samples were collected in 50 ml Eppendorf Conical Tubes (Eppendorf AG, Hamburg, Germany) after light gargling with water. From the polymerase chain reaction (PCR) method analysis, a total of 163 strains were identified in the saliva samples as *S. mutans* (NASH: 88 strains, NAFL: 75 strains). Among the 163 strains, the detection rates for Cnm-positive (Cnm^+^) *S. mutans* and Cnm-negative (Cnm^-^) *S. mutans* were 24.5% and 75.5%, respectively (Cnm^+^: 40 strains, Cnm^-^: 123 strains).

### 2.2 Detection of PA protein and *cnm* and *pac* gene expressions

PA expression was evaluated by western blotting. *S. mutans* strains isolated from overall NAFLD patients were cultured in brain heart infusion broth (BHI; Becton Dickinson, Sparks, MD, USA) at 37 °C for 18 hours. The cultured bacteria were centrifuged at 890 × *g* at 4 °C for 10 minutes, and then, suspended in 100 μl of phosphate-buffered saline (PBS; 137 mM NaCl, 10 mM NaH_2_PO_4_, 8.1 mM Na_2_HPO_4_; Wako Pure Chemical Industries, Osaka, Japan). 80 μl of gel loading buffer [1 M Tris (pH 6.8), 10% sodium dodecyl sulfate (SDS), 0.2% bromophenol blue, 5% glycerol; Wako Pure Chemical Industries] and 20 μl of 10 mM dithiothreitol (DTT) were added, then heat at 100 °C for 10 minutes to prepare the sample for SDS-PAGE. After electrophoresis of the samples using the Mini-PROTEAN Tetra System (Bio-Rad Laboratories, Hercules, CA, USA), proteins were transferred onto a polyvinylidene difluoride (PVDF) membrane (Immobilon; Millipore, Billerica, MA, USA) using a semi-dry blotting system (Trans-blot SD, Bio-Rad Laboratories) at 10 V for 1 hour. The PVDF membrane was incubated overnight at 4 °C in phosphate-buffered saline containing 0.05% Triton X-100 (PBST; Wako Pure Chemical Industries) and 5% skim milk (Becton Dickinson). After washing three times with PBST, the transferred protein bands were reacted with primary antibodies against PA*c* diluted 1:1000 in PBST at room temperature for 1 hour and then washed three times with PBST, and then visualized using an alkaline phosphatase-conjugated antirabbit immunoglobulin G antibody (Dako Denmark A/S, Glostrup, Denmark) diluted 1:1000 in PBST at room temperature for 1 hour. The PVDF membrane was washed three times with PBST after incubation, and then AP buffer containing 5-bromo-4-chloro-3-indolylphosphate-p-toluidine (Wako Pure Chemical Industries) and nitroblue tetrazolium (Wako Pure Chemical Industries) was added to visualize the bands.

Total RNA was extracted from *S. mutans* isolated from NALFD patients and complementary DNA was synthesized by reverse transcription as previously described method (13). Briefly, all *S. mutans* strains were cultured in BHI until the late exponential growth phase (OD_550_ = 0.6), then centrifuged at 890 × *g* at 4 °C for 10 minutes. Total RNA was isolated and treated for 15 minutes at 37 °C with 1.0 U/mL of RNase-free DNase (Amersham Biosciences Corp., Piscataway, NJ, USA) to remove chromosomal DNA. Complementary DNA was synthesized from DNA-free RNA by performing a reverse transcription using SuperScript™ IV Reverse Transcriptase (Invitrogen, Carlsbad, CA, USA) and random primers (Promega Co., Madison, WI, USA). Real-time reverse transcription PCR was performed to evaluate expression levels of *cnm* and the *pac* gene encoding PA using cDNA samples with either 16S rRNA, *cnm*, or *pac* specific primers in an iCycler thermal cycler (Bio-Rad) according with the manufacturer’s protocol. The primer sequences used in this study are listed in **Table 1**. The mRNA expression values were quantified by the ΔΔCT method using 16S rRNA as the internal control.

**Table 1:** PCR primers used in this study.

Name	Sequence (5’-3’)	Purpose	References
16S-F	GTG GGA CGC AAG GAA ACA CAC TGT GC	qRT-PCR	Matsumoto-Nakano & Kuramitsu, 2006 (13)
16S-R	CGT CGC CTT GGT AAG CTC TTA CCT TAC C		
cnm-202F	CTA CCG TTT TCT ACT ATA AGA CTG GGG	qRT-PCR	Naka et al., 2016 (11)
cnm-202R	CCT TCT TGA CCG CGA TAA GAC TCA CTG CCA		
PAC-F	CAC AAA CTG GAA ATC CAG CGA CCA AT	qRT-PCR	This study
PAC-R	ATC TTG GAC AAC ATT GAC ACC AGC AGA		

### 2.3 Assessment of collagen- binding activity

The collagen-binding properties of *S. mutans* were evaluated following the method described by Waterhouse and Russell (14), with some modifications as described previously (15, 16). The collagen-binding properties of each strain were compared with that of the Cnm^+^/PA^+^ *S. mutans* TW871 strain set as 100%, which was shown to cause NASH (10, 17). Briefly, type I collagen [Collagen (type I); Sigma-Aldrich Co., St. Louis, MO, USA] or type IV collagen [Collagen (type IV); Nitta Zelatin Inc., Osaka, Japan] was coated on 96-well microtiter plates for flat bottomed cell culture (FALCON, Franklin Lakes, NJ, USA) and plates were incubated overnight at 4 °C. The plates were washed three times with PBS and blocked for 1.5 h with 5% bovine serum albumin (BSA; Sigma-Aldrich Co.) in PBS containing 0.01% Tween20 (Wako Pure Chemical Industries). *S. mutans* grown overnight in BHI (Becton Dickinson) was collected by centrifugation and added to the wells after the bacterial numbers were adjusted with PBS to OD_550_ = 1.0. After incubation for 3 h at 37 °C, adherent cells were washed three times with PBS and fixed with 200 μL of 25% formaldehyde (Wako Pure Chemical Industries) for 30 minutes at room temperature. After three more washes with PBS, adherent cells were stained with 200 μL of 0.05% crystal violet (Sigma-Aldrich Co., Tokyo, Japan) in water for 1 min and washed three times with PBS. The dye was solubilized by the addition of 7% acetic acid (200 μL) before the determination of OD_595_. Collagen-binding properties of each sample were measured three times and averaged.

### 2.4 *S. mutans* strains

*S. mutans* TW871 (serotype *k*) isolated from the blood of a patient with IE complicated with subarachnoid hemorrhage (18) and KT3 (serotype *e*) isolated from the oral cavity of a patient with severe NASH were used. Both strains were cultured on Mitis-salivarius agar (Becton Dickinson) plates containing bacitracin (0.2 U/ml; Sigma-Aldrich Co.) and 15% (wt/vol) sucrose (MSB agar) or on BHI (Becton Dickinson) broth.

### 2.5 Animal experiments

All animal care and experiments were performed following the National Institute of Health and AERI-BBRI Animal Care and Use Committee guidelines. The study procedures were approved by the Animal Care and Use Committee of Okayama University (Approval No; OKU-2017214) and the Osaka University Graduate School of Dentistry (Approval No; 25-002-0).

We examined the influence of *S. mutans* strains on the development of NASH using a NASH mouse model induced by a high fat diet (HFD) as previously described (10, 11) with some modifications (10, 11, 15). Briefly, 6-week-old C57BL/6J male mice (n =33; Japan SLC, Shizuoka, Japan) were randomly divided into the HFD plus PBS administration group (n=8), HFD plus TW871-treated group (n=9), and HFD plus KT3-treated group (n=16). The sample size was determined on the basis of previous research (10, 11). Mice were allowed free access to water and food throughout the experimental period. HFD 32 (Japan CLEA Inc., Tokyo, Japan), which contains 506.8 kcal/100 g (57.5% from fat, 19.7% from protein, and 22.8% from carbohydrate), was used for the HFD. The levels of saturated and unsaturated fatty acids in the HFD were 22.3% and 76.9%, respectively. Four weeks after beginning the HFD, the *S. mutans* strain TW871 and KT3 (1×10^7^ CFU suspended in 100 μl of PBS) or PBS alone was intravenously injected via the jugular vein. After 12 weeks (16 weeks after beginning the HFD), mice were euthanized, and whole-body weights, liver weights, and visceral fat weights were measured. Euthanasia procedures were conducted in accordance with the AVMA Guidelines for the Euthanasia of Animals (2020). To ensure a humane death with minimal distress, these animals were first anesthetized with a mixture of medetomidine (0.3 mg/kg), midazolam (4 mg/kg), and butorphanol (5 mg/kg) (i.p.) and rendered unconscious. Subsequently, they were administered 100% carbon dioxide (CO_2_) gas at a flow rate of 30–70% of the chamber volume per minute to induce cardiac arrest or respiratory arrest, and CO_2_ gas exposure was continued beyond the onset of respiratory arrest. Whole-body weight increase was calculated based on the whole-body weights at 0 weeks.

Serum samples were collected from the heart at the time of sacrifice and the serum was measured for factors related to liver and bile disease (FALCO Biosystems Ltd., Kyoto, Japan).

Tissue samples were fixed in 10% formaldehyde in PBS, embedded in paraffin, and cut into 3 μm sections for histopathological analysis. Hematoxylin–eosin staining was performed to evaluate the infiltration of inflammatory cells and adipocellular deposition, while Masson’s trichrome staining was used to evaluate fibrosis. Imaging and analyses of the sections were performed following previously reported procedures (19), using microscope images obtained with 200× and 400× objectives (Olympus BX53, Olympus, Tokyo, Japan).

### 2.6 Statistical analysis

Statistical details of experiments are described in the figure legends. GraphPad Prism 8 Statistics Software (GraphPad Inc., La Jolla, CA, USA) were used to perform statistical analysis. A statistical analysis was conducted using Fisher’s exact probability test, Student *t-*test, or Mann-Whitney U test between two groups. *P*-values less than 0.05 was considered statistically significant. A statistical analysis was conducted using ANOVA with Bonferroni’s correction between three groups. *P*-values less than 0.0167 was considered statistically significant.

## 3 Results

### 3.1 Expression of Cnm and PA in *S. mutans* isolated from patients with NAFLD

This study included 40 patients with NAFLD (NASH: 20, NAFL: 20). Patient characteristics are listed in **Table 2**. Alanine aminotransferase and aspartate aminotransferase of patients with NASH were significantly higher compared to those of patients with NAFL (*P* < 0.001). The number of remaining teeth was evaluated as a measure of oral health, which was no significant difference observed between patients with NASH and patients with NAFL (data not shown).

**Table 2:** Subjects’ characteristics.

Characteristics	NASH (n=20)	NAFL (n=20)
Male (%)	8 (40.0)	11 (55.0)
Age (years)	65.7 ± 11.4	66.1 ± 10.4
ALT (IU/mL)	66.1 ± 7.6***	31.2 ± 4.2
AST (IU/mL)	55.7 ± 6.4***	27.2 ± 3.5
BMI (kg/m^2^)	27.0 ± 0.9	26.0 ± 0.8

Data are represented as mean ± SE. ALT, alanine aminotransferase; AST, aspartate aminotransferase; BMI, Body Mass Index. ***P < 0.001 between two groups by Student’s *t*-test.

The detection rate of *S. mutans* in saliva samples from patients with NASH and patients with NAFL was 90% (n=18) and 80% (n=16), respectively, with no significant difference (**Figure 1A**). Among the 163 strains (NASH: 88 strains, NAFL: 75 strains) identified as *S. mutans* by PCR analysis, 62 Cnm^-^/PA^+^ *S. mutans*, 24 Cnm^+^/PA^+^ *S. mutans*, and 2 Cnm^+^/PA^-^ *S. mutans* strains were identified in patients with NASH; no Cnm^-^/PA^-^ *S. mutans* strains were detected in this group. In patients with NAFL, 55, 8, 6, and 6 strains were identified, respectively. There were no significant differences in the detection rates of Cnm^-^/PA^+^ *S. mutans*, Cnm^+^/PA^-^ *S. mutans*, or Cnm^-^/PA^-^ *S. mutans* between the NASH and NAFL groups (**Figure 1B**). However, Cnm^+^/PA^+^ *S. mutans* strains were significantly more prevalent in the NASH group compared with the NAFL group (27% vs. 11%, *P* < 0.05, **Figure 1B**).

**Figure 1:**
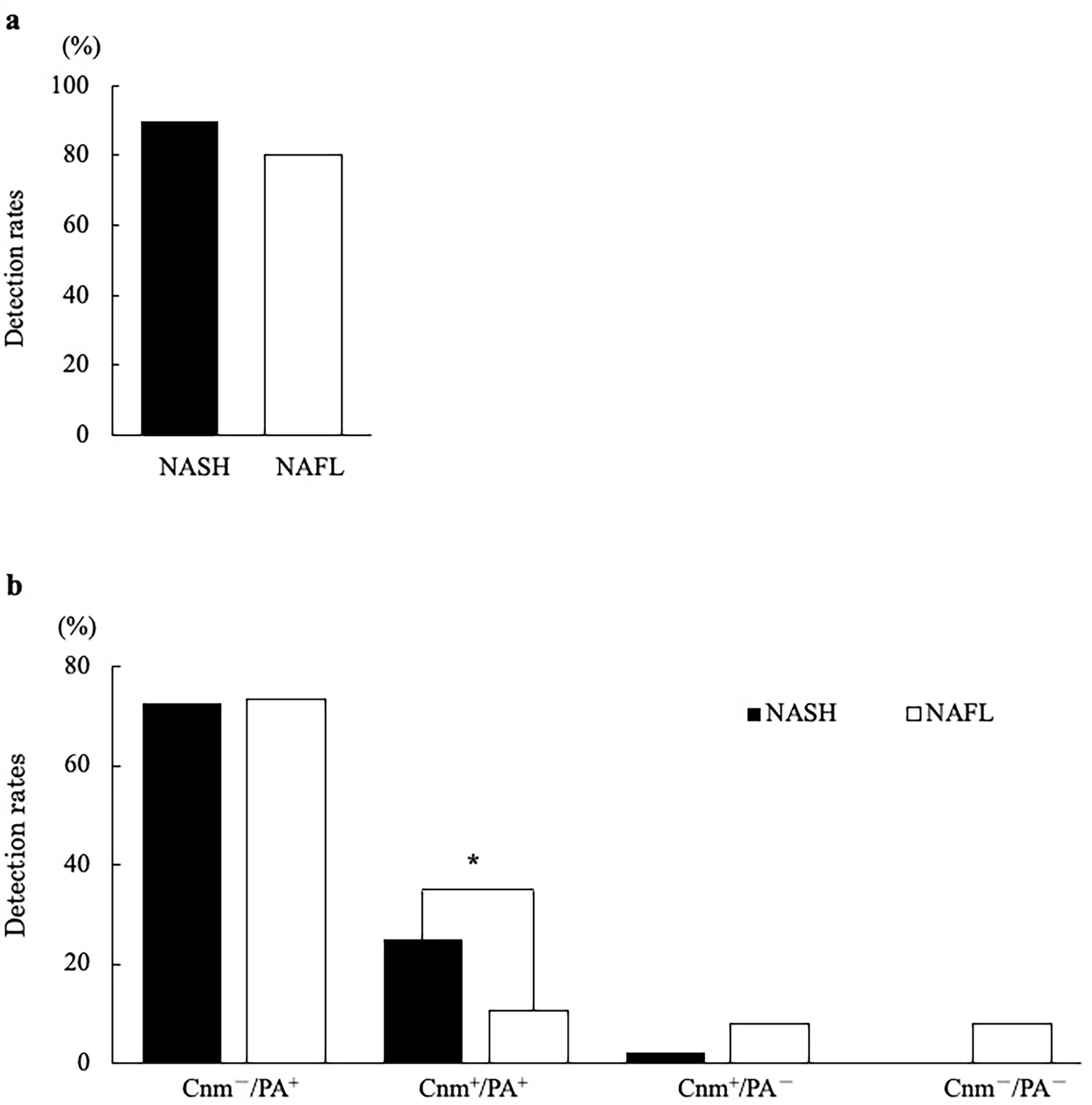
Detection of *S. mutans* strains in patients with NAFLD. Expression of Cnm and PA in *S. mutans* strains isolated and identified from patients with NAFLD (NASH: n=88, NAFL: n=75). There was no significant difference between NASH and NAFL patients regarding the detection rate of *S. mutans* **(A)**. Cnm^+^/PA^+^ *S. mutans* strains were significantly more prevalent in the NASH patients **(B)**. *P < 0.05 between two groups by Fisher’s exact probability test.

Patients with NASH frequently present comorbidities such as obesity, type 2 diabetes mellitus, dyslipidemia, and metabolic syndrome, which can independently alter host immune responses and salivary biochemical composition. To exclude the impact of these variables, we performed multivariable statistical analyses, adjusting for age, sex, hypertension, type 2 diabetes mellitus, and dyslipidemia as independent variables (data not shown). According to the results of the multivariate analysis, when these variables were adjusted for, NASH showed no significant association with these factors. This may be because an insufficient sample size, and further studies with a larger sample size are needed.

### 3.2 The relations between laboratory findings and presence of Cnm^+^/PA^+^ *S. mutans*

Among the 34 patients with NAFLD in whom *S. mutans* was detected (NASH: n=18, NAFL: n=16), 11 patients had Cnm^+^/PA^+^ *S. mutans* (NASH: n=8, NAFL: n=3). We next compared the laboratory findings of patients with NASH with Cnm^+^/PA^+^ *S. mutans* (the “positive group”) and those without Cnm^+^/PA^+^ *S. mutans* (“negative group”). In the positive group, type IV collagen 7S level was significantly higher compared with that of the negative group (*P* < 0.05, **Table 3**). Adiponectin level was significantly lower in the positive group (*P* < 0.05).

**Table 3:** The laboratory findings of NASH with/without Cnm^+^/PA^+^ *S. mutans*.

Characteristics	Positive (n=8)	Negative (n=10)	*P* value
ALT (IU/mL)	49.5 ± 7.5	75.1 ± 11.9	0.052
AST(IU/mL)	43.5 ± 6.0	62.2 ± 10.5	0.083
PLT (×10^4^/μL)	20.8 ± 3.3	19.7 ± 1.6	0.36
FIB4	2.4 ± 0.4	2.2 ± 0.3	0.35
Insulin (μIU/mL)	22.5 ± 8.9	14.4 ± 1.7	0.19
Type IV collagen 7S (ng/mL)	6.6 ± 0.6	5.2 ± 0.4	0.037*
PTX3 (ng/mL)	2.0 ± 0.3	2.2 ± 0.4	0.37
Adiponectin (ng/mL)	6.3 ± 1.1	9.6 ± 0.9	0.022*
IL-6 (pg/mL)	6.0 ± 3.6	2.1 ± 0.2	0.15
TNF-α (pg/mL)	1.2 ± 0.1	1.1 ± 0.1	0.22

Data are represented as mean ± SE. Type IV collagen 7S level was significantly higher, while adiponectin level was significantly lower in NASH with Cnm^+^/PA^+^ *S. mutans* as compared with NASH without Cnm^+^/PA^+^ *S. mutans*. *P < 0.05 between two groups by Student’s *t*-test.

### 3.3 Analysis of the collagen-binding properties of the isolated strains

In the NASH group, the collagen binding ability to type I collagen of the Cnm^+^/PA^+^ *S. mutans* was significantly higher as compared to those of other *S. mutans* (*P* < 0.001, **Figure 2A**). Similar results were shown for type IV collagen (*P* < 0.001, **Figure 2B**). We evaluated the binding ability of Cnm^+^/PA^+^ *S. mutans* in the NASH and NAFL groups to type I and type IV collagen and found significantly higher binding to type IV collagen compared with type I collagen in both groups (*P* < 0.001; NASH, *P* < 0.05; NAFL, **Figure 2C**).

**Figure 2:**
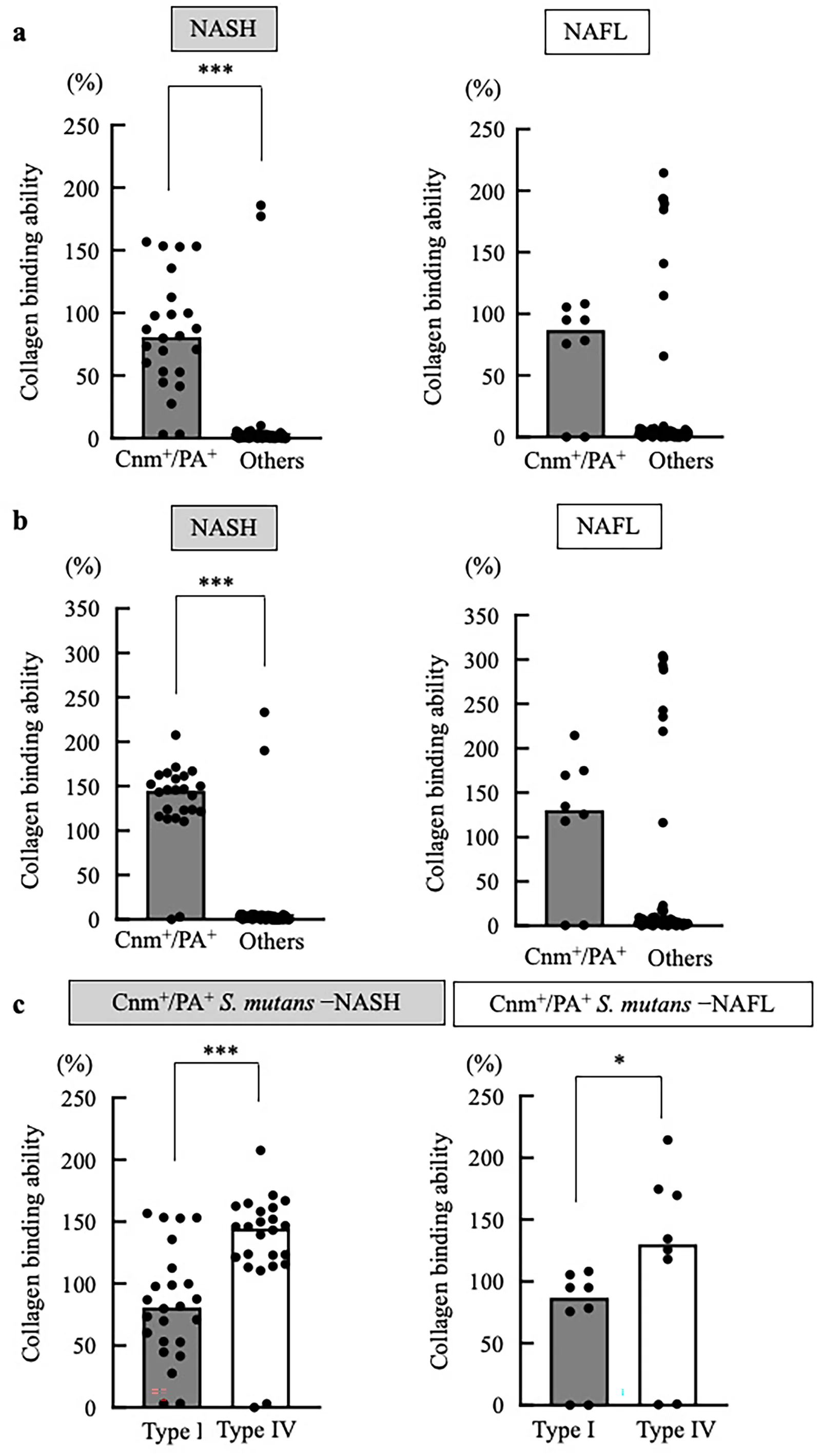
Collagen binding ability of Cnm^+^/PA^+^ *S. mutans* strains in NAFLD patients. In the NASH and NAFL groups, Cnm^+^/PA^+^ *S. mutans* showed significantly higher binding ability to **(A)** type I collagen and **(B)** type IV collagen compared with other *S. mutans* strains. In the NASH and NAFL groups, Cnm^+^/PA^+^ *S. mutans* showed significantly higher binding ability to type IV collagen compared with type I collagen **(C)**. Binding is expressed as the relative percentage standardized to that of TW871 at 100%. Data are shown as the median. *P < 0.05, ***P < 0.001 by Mann-Whitney U test.

### 3.4 Gene expression of *cnm* and *pac* in clinical isolates

We next performed RT-qPCR to evaluate the mRNA expression levels of *cnm* and *pac* in all strains isolated from saliva samples of the overall NAFLD group. We compared the expression levels of *cnm* and *pac*, respectively, in Cnm^+^/PA^+^ *S. mutans* isolated from NASH patients with those in Cnm^+^/PA^+^ *S. mutans* isolated from NAFL patients (**Figures 3a,b**). There was no significant difference between the expression level of *cnm* in Cnm^+^/PA^+^ *S. mutans* isolated from NASH and NAFL groups (**Figure 3A**). However, the expression level of *pac* was significantly higher in Cnm^+^/PA^+^ *S. mutans* from the NASH group (*P* < 0.05, **Figure 3B**). We further compared *pac* expression levels in Cnm-positive and Cnm-negative *S. mutans*, both of which were isolated from NASH patients or NAFL patients (**Figures 3C, D**). In the NASH group, the expression level of *pac* in *S. mutans* with Cnm was significantly higher than that in *S. mutans* without Cnm; no differences were observed in the NAFL group (*P* < 0.05, **Figures 3C, D**).

**Figure 3:**
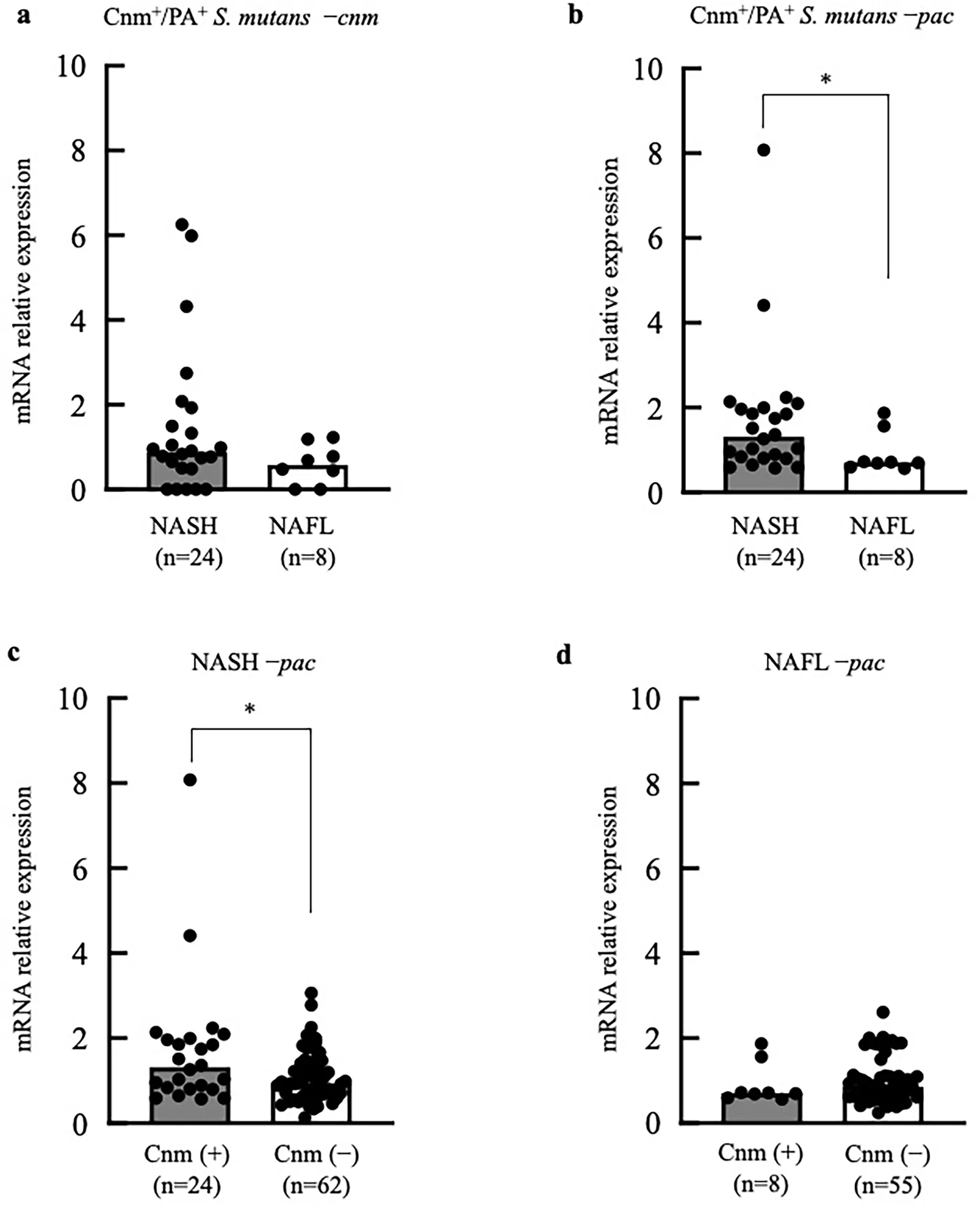
mRNA expression levels of *cnm* and *pac* of Cnm^+^/PA^+^ *S. mutans* in NASH and NAFL. There was no significant difference in the expression levels of *cnm* in Cnm^+^/PA^+^ *S. mutans* in NASH and NAFL **(A)**. However, the expression level of *pac* was significantly higher in NASH than NAFL **(B)**. In patients with NASH, the expression level of *pac* was significantly higher in Cnm^+^/PA^+^ *S. mutans* than that in *S. mutans* without Cnm **(C)**. In contrast, no difference was observed in patients with NAFL **(D)**. mRNA expression is expressed as the relative number standardized to that of TW871 at 1.0. Data are shown as the median. *P < 0.05 by Mann-Whitney U test.

### 3.5 *S. mutans* induced NASH aggravation in NASH model mice

We next examined the effects of intravenous administration of *S. mutans* strains on the development of NASH using a NASH mouse model. We induced NASH using a HFD for 4 weeks, followed by administration of *S. mutans* TW871 isolated from a patient with IE or KT3 isolated from a patient with severe NASH. PBS served as the control. Mice continued with the HFD for an additional 12 weeks. After 16 weeks, there were no differences in body mass or visceral fat among the groups (**Figures 4A, B**). However, liver weight was significantly greater in the KT3 groups compared with the non-treated groups (*P* < 0.0167, **Figure 4C**).

**Figure 4:**
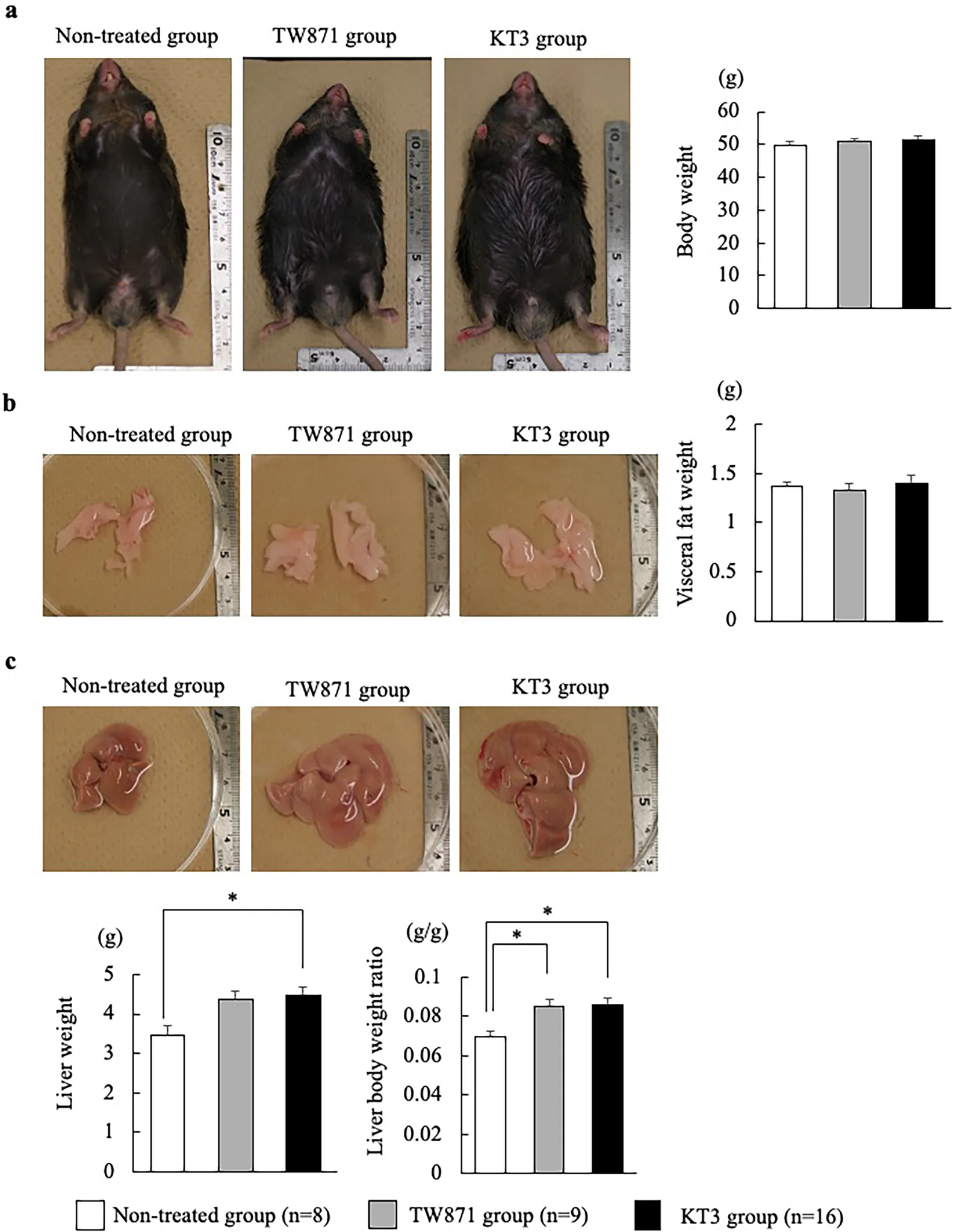
Impact of TW871 and KT3 on aggravation of NASH in model mice. There were no differences regarding body mass or visceral fat size among the groups **(A, B)**. Liver weight was significantly greater in the KT3-treated groups compared with the non-treated group **(C)**. Each column represents the mean ± SE from 8–16 animals. Statistical significance was determined using analysis of variance (ANOVA) with Bonferroni’s correction; *P < 0.0167.

After 16 weeks, the mice treated with TW871 and KT3 strains showed significantly increased levels of serum total cholesterol, free cholesterol, LDL cholesterol, HDL cholesterol, and cholinesterase factors that are related to abnormal lipid metabolism (*P* < 0.0167, **Figure 5A**). We also observed significantly increased levels of serum aspartate aminotransferase, alanine aminotransferase, alkaline phosphatase, leucine aminopeptidase, and serum iron, which are related to liver dysfunction (*P* < 0.0167; ALP, LAP, Fe TW871/KT3 vs. Non-treated group, AST TW871 vs. Non-treated group, ALT KT3 vs. Non-treated group, **Figure 5B**).

**Figure 5:**
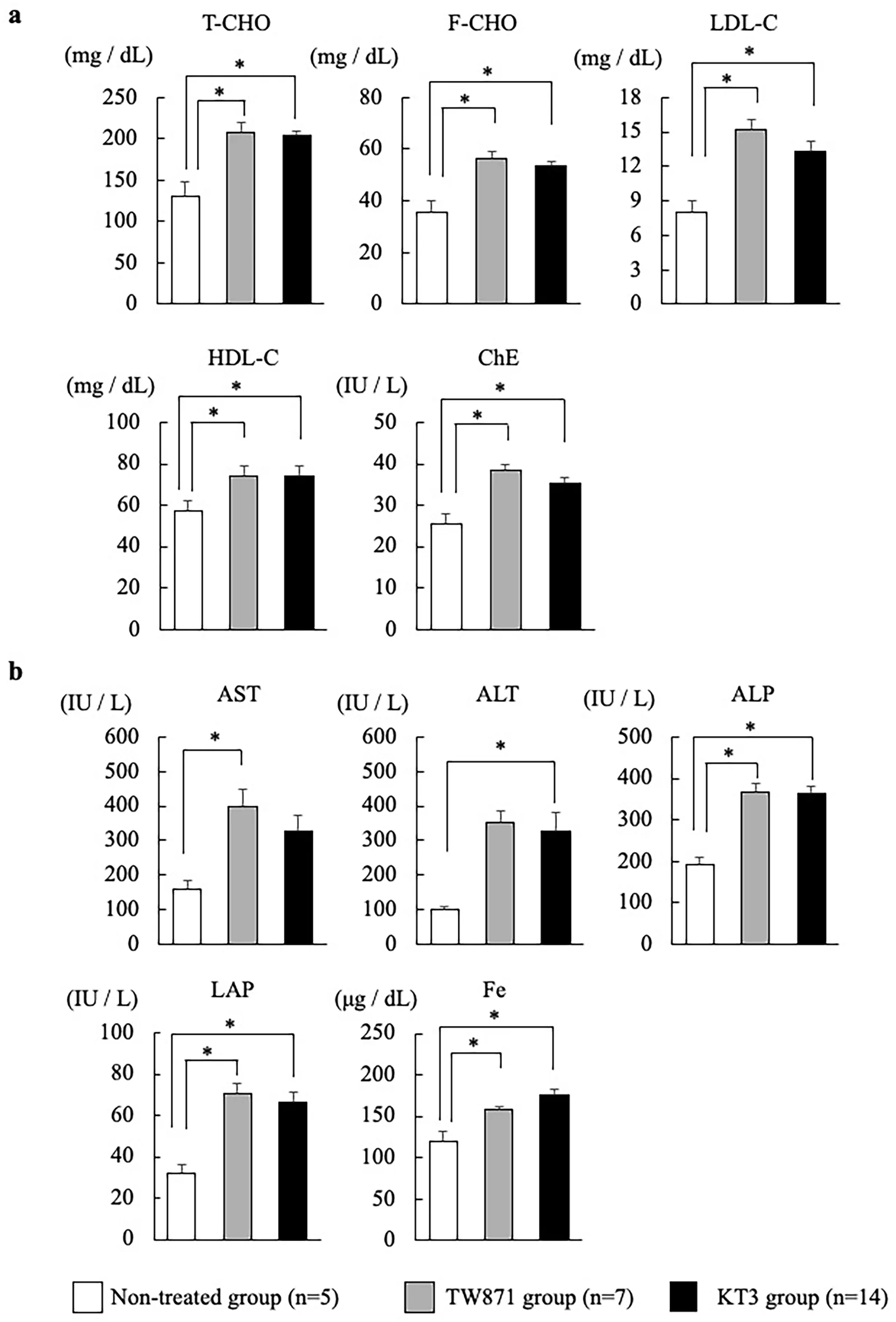
Effect of *S. mutans* administration on serum biochemical indicators in NASH model mice. Serum levels of **(A)** lipid metabolism markers including total cholesterol (T-CHO), free cholesterol (F-CHO), LDL cholesterol (LDL-C), HDL cholesterol (HDL-C), and cholinesterase (ChE) and **(B)** hepatic dysfunction markers including aspartate aminotransferase (AST), alanine aminotransferase (ALT), alkaline phosphatase (ALP), leucine aminopeptidase (LAP), and serum iron (Fe) were significantly increased compared with levels in the non-treated group. Each column represents the mean ± SE from 5–14 different animals. Statistical significance was determined using ANOVA with Bonferroni’s correction; *P < 0.0167.

The histopathological appearance of NASH is characterized by macrovesicular steatosis of hepatocytes, as well as intralobular infiltration of inflammatory cells such as neutrophils and lymphocytes, hepatocellular ballooning, and fibrosis. Lipid accumulation was observed in more than 5% of the hepatocytes in all groups: the non-treated group, the TW871 group, and the KT3 group. However, HE staining of liver sections from mice after 16 weeks revealed marked lipid accumulation and lobular inflammation around hepatocytes and blood vessels in both the KT3 and TW871 groups; these changes were not observed in the non-treated group (**Figure 6a**). The KT3 and TW871 groups showed lager droplets of adipose deposition than the non-treated group, and also fusion of lipid droplets was observed in the KT3 and TW871 groups. (**Figure 6A**). Furthermore, hepatocellular ballooning was observed in the HE staining sections of the KT3 and TW871 groups (**Figure 6A**). In the Masson’s trichome-stained section, prominent fibrosis with blue staining was observed in the liver parenchyma of the KT3 and TW871 groups, which was not observed in the non-treated group (**Figure 6B**). In the KT3 group, similar to the TW871 group, fibrosis was observed around blood vessels and hepatocytes; however, marked fibrosis was observed in the KT3 group, with collagen accumulation surrounding individual hepatocytes (pericellular fibrosis).

**Figure 6:**
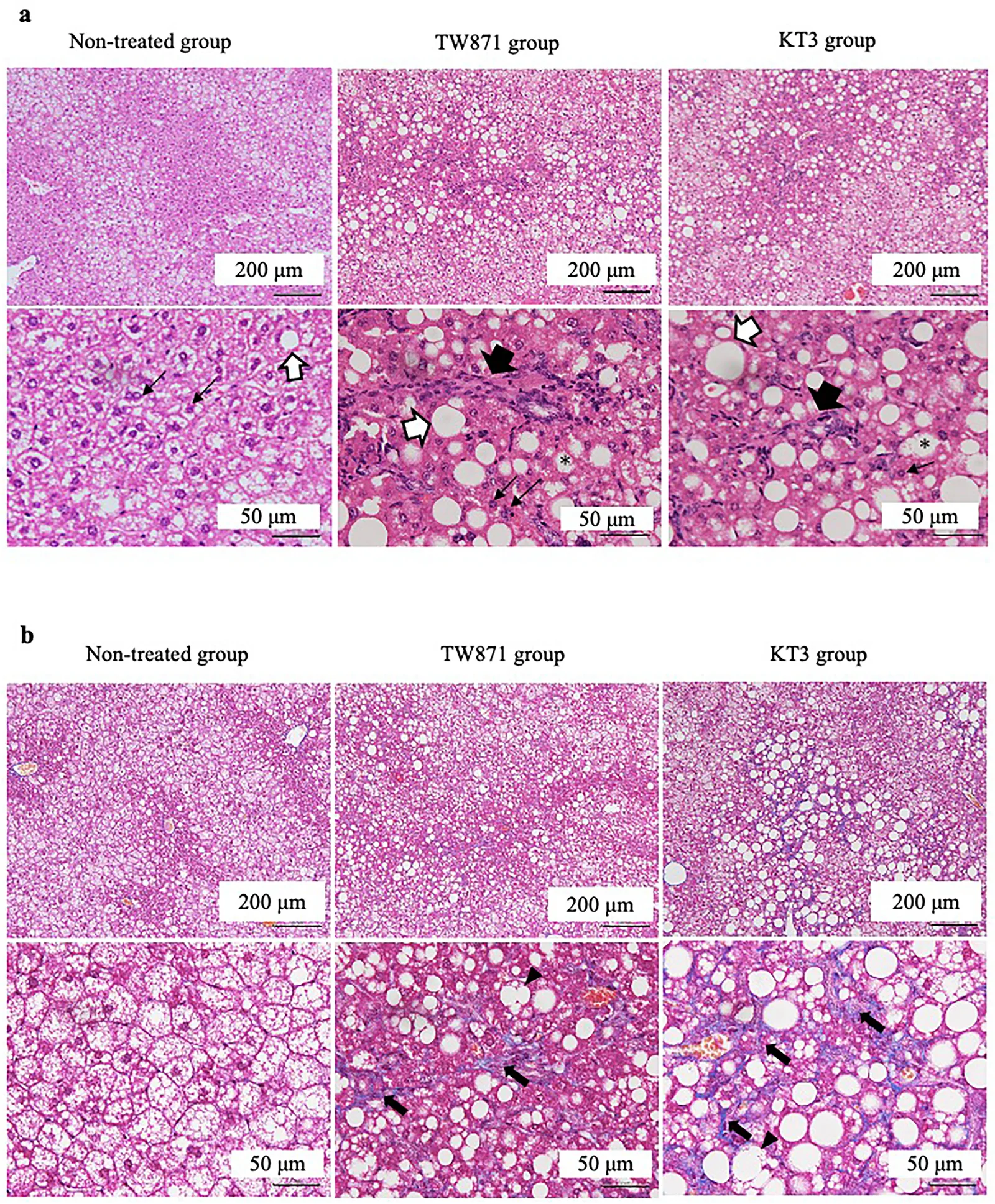
Representative histopathological images of liver tissues in NASH model mice administered with *S. mutans*. Liver tissues were evaluated after 16 weeks (4 weeks of high-fat diet, followed by *S. mutans* or PBS with high-fat diet for 12 weeks). Bottom panels are higher magnification of top panels. HE staining revealed marked lipid accumulation in liver tissues from all groups, with lobular inflammation observed in both the KT3 and TW871 groups **(A)**. The open and closed arrows indicate adipose deposition and lobular inflammation, respectively. Pointed arrows indicate hepatocytes. MT staining showed marked fibrosis in liver tissues from the KT3 and TW871 groups **(B)**. The dark arrows indicate fibrosis. Scale bars: 200 μm (top panels) and 50 μm (bottom panels).

## 4 Discussion

Cnm-positive *S. mutans* strains exhibit high collagen-binding activity and a tendency for a low level of glucosyltransferase and glucan-binding protein activities, which are related to causing dental caries (20). Additionally, Cnm-positive *S. mutans* strains may be more likely to be carried in the blood stream compared with Cnm-negative strains (17). Several studies reported that Cnm-positive *S. mutans* strains may be related to the occurrence of IE, hemorrhagic stroke, and ulcerative colitis since Cnm-positive *S. mutans* strains show high collagen-binding activities and high resistance to phagocytosis (1–4). In our previous study, the prevalence of Cnm-positive *S. mutans* strains was significantly higher in patients with NASH among NAFLD (12). Therefore, we speculated that Cnm-positive *S. mutans* strains may aggravate NASH from NAFL.

The “two-hit theory” is widely accepted as the mechanism of NASH, in which insulin resistance because of metabolic syndrome caused by excessive nutritional intake results in diabetes, atherosclerosis, and dyslipidemia, and NAFL (the first step). Oxidative stress, lipid peroxidation, and mitochondrial dysfunction, which induce inflammatory changes and fibrosis in the liver, (as the second step), induce NASH (21–23). Recently, the “multiple parallel-hit theory” has been widely supported, in which the interaction between the liver and extrahepatic organs such as adipose tissue and intestinal tract contributes to the development of NASH (24). In our previous study using a mouse model in which fat accumulation was induced in the liver by feeding a HFD, Cnm^+^/PA^+^ *S. mutans* was shown to adhere to hepatocytes in the presence of unsaturated fatty acids by binding to hepatocytes via Cnm and to unsaturated fatty acids via PA, resulting in increased fibrosis, which may be involved in the pathological progression from NAFL to NASH (11). Intravenous administration of Cnm^+^/PA^+^ *S. mutans* to 6-week-old C57BL/6J mice fed a HFD induced NASH-like condition at 12 weeks, suggesting that Cnm^+^/PA^+^ *S. mutans* is involved in the aggravation of NASH (10). Furthermore, we observed that mice that were intravenously administered a *S. mutans* strain with a defect in Cnm or PA or a Cnm and PA double knockout strain did not show clinical or histopathological signs of NASH aggravation, while infection with either of the complemented strains reproduced the aggravated NASH conditions in our previous study (11). Therefore, in this study, we focused on Cnm and PA. While the prevalence of *S. mutans* strains was similar in patients with NASH and patients with NAFL, the prevalence of Cnm^+^ and PA^+^ *S. mutans* strains among all *S. mutans* strains was higher in patients with NASH compared with those with NAFL (27% vs. 11%). The prevalence of Cnm^+^ and PA^+^ *S. mutans* strains in the patients with NASH was previously reported as 7.5% (5). Therefore, this strain was detected at a higher frequency than usual in patients with NASH.

Analysis of laboratory findings revealed that type IV collagen 7S was higher in patients with NASH with Cnm^+^ and PA^+^ *S. mutans* strains compared with patients with NASH with other strains, while adiponectin showed the opposite trend. The type IV collagen 7S domain, which is involved in connective tissue metabolism, has been identified as a biochemical marker for assessing fibrogenesis and the severity of fibrosis in patients with cirrhosis (25). The increase in type IV collagen 7S level correlated with the amount of fibrosis and increase in synthesis from fibroblasts following increasing fibrosis in the liver (25). Because it increases in the blood relatively early during liver fibrosis, it is useful as an indicator of liver fibrosis. For these reasons, it is also expected to serve as a marker for distinguishing NAFL from NASH, potentially avoiding the need for liver biopsy (26). Adiponectin is one of the adipocytokines, which are bioactive substances specifically secreted by adipocytes; it decreases during obesity and visceral fat accumulation while increasing with weight loss (27). Administering adiponectin to mice fed a HFD has been reported to enhance the clearance of free fatty acids in the blood and improve glucose metabolism (28). Thus, adiponectin is closely associated with adipose and hepatic tissues. In this study, adiponectin levels were significantly reduced in the NASH group harboring Cnm^+^/PA^+^ *S. mutans.* Previous studies have reported that among various fatty acids, oleic acid and linoleic acid (unsaturated fatty acids) exhibit significantly higher binding to PA^+^ *S. mutans* strains compared with PA^-^ *S. mutans* strains (11). Adipose tissue consists of subcutaneous fat located beneath the skin and visceral fat found around internal organs, with the fatty acid composition primarily consisting of unsaturated fatty acids. Additionally, adiponectin is secreted in greater amounts from visceral fat cells compared with subcutaneous fat cells (29). Therefore, it is suggested that the decrease in adiponectin secretion may have impaired lipid metabolism in visceral fat and promoted fat accumulation.

Inflammatory cytokines such as TNF-α and IL-6, as well as oxidative stress, are involved in adiponectin secretion, and these factors have been shown to suppress adiponectin expression (30). However, in this study, while a significant decrease in adiponectin was observed in the NASH patient group harboring Cnm^+^/PA^+^ *S. mutans* compared with the group with other strains, no difference in TNF-α or IL-6 expression was observed. Previous studies using DNA microarray analysis showed that IFN-γ and metallothionein were significantly upregulated in mouse livers 24 h after TW871 strain administration (15). IFN-γ is produced by activated T lymphocytes and NK cells, activates macrophages, and induces the secretion of inflammatory cytokines such as TNF-α, IL-6, and TGF-β. Plasma concentrations of TNF-α and IL-6 have been reported to peak at 1.5 h and 2 h after administration, respectively (31), therefore, it is necessary to investigate the dynamics of various inflammatory cytokines over different experimental periods in future studies.

The *S. mutans* surface protein Cnm possesses collagen-binding ability (32). We evaluated Cnm expression in *S. mutans* strains isolated from patients with NAFLD using a collagen-binding assay. Cnm^+^/PA^+^ *S. mutans* isolated from patients with NASH showed significantly higher binding capacity to both type I and type IV collagen compared with Cnm^+^/PA^−^ *S. mutans*, Cnm^−^/PA^+^ *S. mutans*, and Cnm^−^/PA^−^ *S. mutans*, which do not express both proteins. Furthermore, Cnm^+^/PA^+^ *S. mutans* isolated from NAFL and NASH groups bound significantly more strongly to type IV collagen than to type I collagen. These findings indicate that Cnm^+^/PA^+^ *S. mutans* strongly binds to type IV collagen. Type IV collagen is present in the basement membrane of capillaries located between hepatocytes in liver sinusoids; however, the basement membrane structure is absent in normal liver sinusoids (33). During liver fibrosis, basement membrane components are secreted from hepatic stellate cells within the sinusoids and deposited, leading to basement membrane formation. This results in elevated levels of type IV collagen and type IV collagen 7S in liver tissue and blood (34). Therefore, we speculate that Cnm^+^/PA^+^ *S. mutans* may bind to liver tissue where basement membrane components are deposited and may be involved in liver fibrosis.

Previous reports have suggested that Cnm and PA may be involved in systemic diseases, while no studies have examined their expression levels of Cnm and PA. In this study, we analyzed the expression levels of *cnm* and *pac* in *S. mutans* strains isolated from NAFLD patients using RT-qPCR. The expression level of *pac* in Cnm^+^/PA^+^ *S. mutans* from patients with NASH was significantly higher compared with that in patients with NAFL. Furthermore, the expression level was significantly higher in *S. mutans* strains possessing *cnm* compared with those lacking *cnm*. These findings suggest that *pac* expression is involved in NASH progression and that its expression level may correlate with *cnm* expression.

We next administered Cnm^+^/PA^+^ *S. mutans* isolated from patients with NASH to a HFD-induced insulin-resistant NASH mouse model. The specimens infected with Cnm^+^/PA^+^ *S. mutans* clearly exhibited features consistent with NASH, as demonstrated by biochemical and histological analysis. Additionally, increased production of type IV collagen in liver tissue was observed. Binding of *S. mutans* to type IV collagen via Cnm may contribute to liver fibrosis, potentially leading to NASH development. Previous reports have shown that a decrease in the production of N-acyl ethanolamine—a key mediator of metabolic homeostasis and inflammation—in adipose tissue induces impaired intestinal barrier function and dysbiosis of the gut microbiota, which in turn contributes to the metabolic abnormalities observed in adipose tissue (35). Additionally, it has been reported that intestinal inflammation can alter the host’s immune response toward oral bacteria, promoting oral dysbiosis and exacerbating local inflammation (36). Therefore, the possibility that systemic changes associated with NASH may selectively shape or alter the oral microenvironment, thereby affecting bacterial profiles. The abundance of *S. mutans* and the expression of its virulence genes are directly influenced by active dental caries, chronic periodontitis, and oral hygiene practices. In this study, the number of remaining teeth was evaluated as a measure of oral health (data not shown). No significant difference was observed between patients with NASH and patients with NAFL in this regard, and thus it is possible that the differences observed in this study were not simply the result of a deterioration in baseline oral health among patients with NASH. Nevertheless, a detailed investigation regarding the oral health status among patients with NAFLD in future studies is required. Additionally, further research is necessary regarding the oral microbiota, including *S. mutans*, and its association with NASH.

## Data Availability

The original contributions presented in the study are included in the article/supplementary material. Further inquiries can be directed to the corresponding author/s.
